# Advancements in Endoscopic Resection for Colitis-Associated Colorectal Neoplasia in Inflammatory Bowel Disease: Turning Visible into Resectable

**DOI:** 10.3390/diagnostics14010009

**Published:** 2023-12-20

**Authors:** Roberta Maselli, Roberto de Sire, Davide Massimi, Gianluca Franchellucci, Anita Busacca, Fabiana Castiglione, Antonio Rispo, Cesare Hassan, Alessandro Armuzzi, Alessandro Repici

**Affiliations:** 1 Gastroenterology, Endoscopy Unit, IRCCS Humanitas Research Hospital, 20089 Rozzano, Italy; roberta.maselli@hunimed.eu (R.M.); davide.massimi@humanitas.it (D.M.); cesare.hassan@hunimed.eu (C.H.); alessandro.repici@hunimed.eu (A.R.); 2 Department of Biomedical Sciences, Humanitas University, 20072 Pieve Emanuele, Italy; gianluca.franchellucci@humanitas.it (G.F.); alessandro.armuzzi@hunimed.eu (A.A.); 3 IBD Unit, Department of Clinical Medicine and Surgery, University Federico II, 80126 Naples, Italy; fabcasti@unina.it (F.C.); antonio.rispo2@unina.it (A.R.); 4 Gastroenterology, IBD Unit, IRCCS Humanitas Research Hospital, 20089 Rozzano, Italy; anita.busacca@humanitas.it

**Keywords:** inflammatory bowel disease, IBD, dysplasia, neoplasia, surveillance, endoscopic resection, endoscopic mucosal resection, EMR, endoscopic submucosal dissection, ESD

## Abstract

Patients suffering from inflammatory bowel disease (IBD) face a two to three-fold higher risk of developing colorectal cancer (CRC) compared to the general population. In recent years, significant progress has been made in comprehending the natural history of IBD-associated CRC (IBD-CRC) and refining its treatment strategies. The decreased incidence of IBD-CRC can be attributed to improved therapeutic management of inflammation, advancements in endoscopy, and early detection of precancerous lesions via surveillance programs. Advanced imaging technologies have made previously undetectable dysplasia visible in most cases, allowing for a much more precise and detailed examination of the mucosa. Additionally, new tools have facilitated the endoscopic resection (ER) of visible lesions in IBD. Particularly, the key to effectively manage colitis-associated colorectal neoplasia (CAN) is to first identify it and subsequently guarantee a complete ER in order to avoid surgery and opt for continuing surveillance. Advanced ER techniques for CAN include endoscopic mucosal resection (EMR), endoscopic submucosal dissection (ESD), and hybrid ESD-EMR (h-ESD). This narrative review aims to consolidate the current literature on IBD-CRC, providing an overview of advanced techniques for ER of CAN in IBD, with a particular emphasis on the impact of ESD on the long-term outcomes of IBD patients.

## 1. Introduction

Inflammatory bowel disease (IBD), including ulcerative colitis (UC) and Crohn’s disease (CD), represents chronic and relapse-remitting inflammatory disorders, resulting from a complex interplay among genetic predisposition, gut microbiota (GM), and environmental factors, leading to the dysregulation of the immune system [1,2,3,4]. IBD patients face an elevated risk of developing dysplasia and colorectal cancer (CRC) [5,6,7]. The molecular pathways involved in IBD-associated CRC (IBD-CRC) exhibit distinctions from those identified in sporadic CRC (sCRC) [5]. In sCRC, the predominant pathway is the adenoma-to-carcinoma sequence, whereas in IBD-CRC, the sequence typically involves chronic inflammation leading to dysplasia and, ultimately, carcinoma [8]. Specifically, individuals with long-standing UC and Crohn’s colitis (excluding limited proctitis) experience a two to three-fold increased risk of CRC [9,10]. The exact risk varies depending on several individual factors, including the extension of inflammation, a family history of CRC, the presence of primary sclerosing cholangitis (PSC), male gender, and younger age at the time of diagnosis [11]. Additionally, other factors associated with more severe disease include a high inflammatory burden, backwash ileitis, pseudopolyps, prior dysplasia, and colonic strictures [12].

Fortunately, there is a positive trend showing a decline in the rates of colitis-associated colorectal neoplasia (CAN) over time [13]. This decline is likely attributed to the enhanced control of inflammation through improved medical therapies, as well as advancements in endoscopic techniques for detecting and removing precancerous lesions, resulting in the enhancement of surveillance programs [14]. Advanced imaging technologies have made previously undetectable dysplasia visible in most cases, allowing for a much more precise and detailed examination of the mucosa. Additionally, new tools have facilitated the endoscopic resection of visible lesions, enabling patients to avoid colectomy. Nevertheless, CAN continues to be a leading cause of mortality and a primary driver for colectomy in the IBD population [15]. In fact, a substantial portion of colorectal dysplasia in IBD patients presents as non-polypoid and can be challenging to detect [16]. According to American and European guidelines, it is recommended to consider endoscopic resection (ER) for cases of visible and endoscopically resectable colorectal dysplasia in patients with IBD [7,17,18,19]. However, ER of CAN, particularly with larger lesions, can pose challenges attributable to persistent inflammation, scarring of the mucosa, and fibrotic changes in the submucosa [20].

Here, we aim to summarize the existing literature on IBD-CRC, providing an update on advanced endoscopic resection techniques for CAN with a particular focus on the impact of endoscopic submucosal dissection (ESD) on the longitudinal outcomes of IBD patients.

## 2. Literature Research Method

Authors conducted an electronic database search using PubMed and Medline, covering the period from inception to October 2023. The search utilized the terms “ulcerative colitis” OR “UC”, OR “Crohn’s disease” OR “CD”, OR “inflammatory bowel disease” OR “IBD”, AND “dysplasia” OR “neoplasia”, OR “colitis-associated dysplasia” OR “colitis-associated neoplasia”, AND “endoscopic mucosal resection” OR “EMR”, OR “endoscopic submucosal dissection” OR “ESD”. 

A supplementary search was conducted on the basis of the references of the selected papers. We screened the articles for suitability for the scope of this narrative review, then reviewed the full text of articles and excluded those that did not fit the aim of our paper.

## 3. Carcinogenesis in Inflammatory Bowel Disease

**Figure 1:**
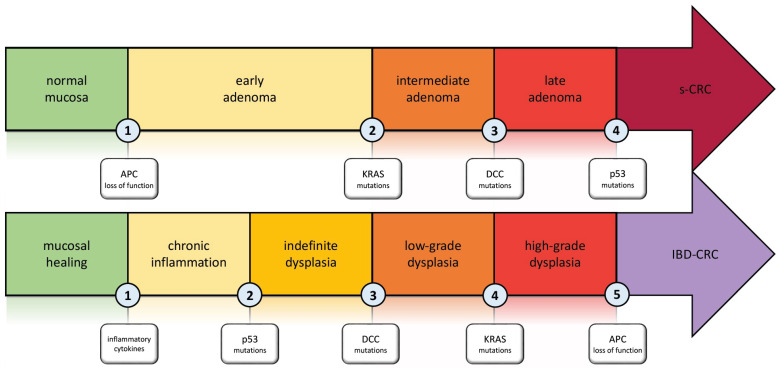
Carcinogenesis in Inflammatory Bowel disease: the inflammation–dysplasia–carcinoma cascade compared to the adenoma–carcinoma cascade involved in s-CRC. Abbreviations. APC: adenomatous polyposis coli; KRAS: Kirsten rat sarcoma viral oncogene homolog; DCC: deleted in colon cancer; s-CRC: sporadic colorectal cancer; IBD-CRC: inflammatory bowel disease-associated colorectal cancer.

The typical pathogenesis pattern in sCRC is the adenoma–carcinoma sequence. In IBD-CRC, this concept has been further developed and adapted to the different setting (Figure 1) [21,22,23]. Carcinogenesis in IBD is the result of multiple factors such as chronic inflammation, genetics, epigenetic modifications, and changes in the gut microbiome that drive the transition from normal mucosa to malignancy [24,25]. The key differences between the pathogenesis of sCRC and IBD-CRC, as well as the particular pattern of IBD-CRC development, are discussed in this section. 

Using multi-region exome sequencing of fresh frozen samples, Baker et al. [25] quantified, for the first time, the intratumor genetic heterogeneity in IBD-CRC. They also demonstrated that the divergence of the molecular pattern from sCRC and IBD-CRC begins in the non-dysplastic colonic mucosa, far from the formation of an identifiable lesion. The IBD-CRC sample analyzed in the study had a burden of 3.0 single nucleotide alterations (SNA) per Mb, which is 20% higher than sCRC [26]. The SNA burden mutations were found to be highly prevalent in non-dysplastic tissue and were also dominated by age-associated signatures, suggesting that inflammation and injury-induced cell turnover required for intestinal repair led to this level of SNA [26]. One genetic alteration that distinguishes IBD-CRC from sCRC is early Tp53 mutation. This type of alteration typically occurs late in sCRC but is present not only in precancerous dysplasia, but also in non-dysplastic mucosa [27,28,29]. Aneuploidy is one of the early characteristics of colitis-associated colorectal precancerous lesions. It strongly correlates with dysplasia and CRC, as initially demonstrated by Rubin et al. [30] and subsequently confirmed during a 9.2-year follow-up [31]. A further distinction of IBD-CRC is the infrequent occurrence of APC gene mutations. The difference in mutations was statistically significant when comparing the specimens from IBD patients with data from genomic datasets TCGA-CRC [32] and FM-CRC [33]. K-ras alterations were observed less frequently in IBD-CRC compared to sCRC in several studies [26,33,34]. Therefore, C-my amplifications appear to be more common in IBD-CRC than in sCRC series [35].

As previously summarized, while APC is less frequently mutated in CAC, especially in the early stages, SOX9, an antagonist of Wnt/beta-catenin signaling, appears to suffer from significant levels of loss of function alterations, resulting in a push towards a tumorigenesis path [36]. These data attest to a Wnt-alteration bypassing APC, which may ultimately be driven by other triggers such as chronic inflammation [36]. It should be noted that chronic inflammation is a significant factor in the development of cancer in individuals with IBD. Inflammation can lead to cancer through direct or indirect pathways. The direct pathway involves oxidative stress and subsequent DNA damage, while the indirect pathway is linked to cytokines produced by inflammatory and intestinal cells [26,37,38]. The excess of oxidative stress leads to the activation of DNA damage response (DDR) that includes mechanisms involved in the regulation of the cellular cycle and senescence process. For indirect damage, multiple mechanisms should be considered. TNF is well known as one of the main factors in IBD, and these signaling pathways are correlated with factors implicated in the regulation of the cellular cycle and DDR [39]. For instance, TNF Receptor 2 (TNFR2) activation induces caspase-3, leading to up-regulation of myosin light chain kinase and subsequent release of pro-tumorigenic cytokines, and breakdown of tight junctions [37,40]. Moreover, the STAT3 pathway is involved in carcinogenesis in IBD patients [41,42]. STAT3’s role in IBD-CRC was observed in the murine model, where its inactivation led to a reduction in tumor growth [43]. The pathogenesis of IBD-CRC must acknowledge the influence of gut microbiome (GM) alterations. The GM has played an important role in CRC research over the past two decades, with variations in its quality and quantity being associated with the development of both sCRC and IBD-CRC [44]. In addition, GM is a requirement for the development of spontaneous adenocarcinoma in T-cell receptor beta chain and p53 double-knockout mice [45]. Toll-like receptors (TLR) are expressed extensively by intestinal epithelial cells; however, TLR4 was observed to be overexpressed in CRC specimens derived from patients with UC [46,47]. TLR4 overexpression was associated with the upregulation of dual oxidase 2 (DUOX 2) and NADPH oxidase, resulting in an increase in hydrogen peroxide (H_2_O_2_) and oxidative stress. This increase caused alterations in the bacterial population [47]. No changes were needed as the text already adheres to the given principles. IBD-CRC was diagnosed in patients with reduced Firmicutes/Bacteroides [48]. Additionally, a reduction in Clostridium butyricum was estimated to positively impact IBD-CRC development. Clostridium butyricum stimulated BLC2, supporting its pro-apoptotic nature, and appeared to decrease IL-6 and TNF-a levels [48]. Lastly, recent studies have implicated Fusobacterium nucleatum in the pathogenesis and progression of CRC through various mechanisms. For example, it binds to the PD-L1 receptor, a well-known immune checkpoint that promotes tumor immune escape; it also causes depletion of T cells and enrichment of depleted CD8+ in the tumor microenvironment; and it induces epithelial-mesenchymal transition (EMT) characteristics of CRC both in vitro and in vivo models [49]. Multiple and different factors are involved in IBD-CRC pathogenesis and differentiate itself from sCRC. This specific feature could be useful for detection, prognostic determination, and even therapeutic target in future clinical application [50]. 

Regarding the anatomical distribution of CRC in UC, the majority of authors report a prevalence in the left-sided colon [51]. This aligns with the usual localization of inflammation in UC patients, starting in the rectum and extending proximally. Nevertheless, different studies have identified varying CRC localizations in CD. For instance, Choi et al. documented a heightened incidence of tumors in the right colon [51], while Svrcek et al. noted a predominance in the rectum and sigma [52]. A noteworthy dissimilarity in the anatomical distribution of tumors between CD and UC was observed in a recent study aimed at assessing the clinicopathological features and outcomes of patients with CRC, comparing UC with CD [53]. Patients with Crohn’s colitis exhibited a significant prevalence of rectal cancers, consistent with prior studies, possibly attributed to active inflammation with fistulas in this region for most patients [53]. In contrast, a substantial number of UC patients had a CRC in the right-sided colon [53].

## 4. Surveillance in Inflammatory Bowel Disease

### 4.1. Indication and Timing of Endoscopic Surveillance

Patients with long-standing colitis face a heightened risk of developing CRC compared with the general population, with a cumulative risk <5% after 10 years, <10% at 20 years, and ~20% at 30 years from the onset of the disease [54]. According to latest ECCO-ESGAR Diagnostics Guidelines [55], it is recommended to provide screening colonoscopy to all patients 8 years after the onset of symptoms in order to reevaluate disease progression and rule out dysplasia/CRC. The recognized risk factors influencing the onset of IBD-CRC include the disease duration and the extent, persistent uncontrolled inflammation, a concurrent PSC, and a family history of CRC [56]. Continuous surveillance is recommended for all patients, except those with proctitis where the disease is confined to the rectum without any indication of past or present endoscopic or microscopic inflammation proximal to the rectum. In such cases, participation in a routine surveillance colonoscopy program is not deemed required. In patients with concurrent PSC, on the other hand, annual endoscopy should be completed, regardless of IBD characteristics. The pre-established intervals of endoscopic surveillance according to ECCO guidelines [55], therefore, are stratified by colon cancer risk as follows: (A) lower risk—colonoscopy every 5 years for extensive colitis with mild endoscopic or histological inflammation and colitis affecting <50% of the colon; (B) intermediate risk—colonoscopy every 2–3 years for extensive colitis with mild endoscopic or histological activity and a first-degree relative >50 years with CRC; (C) higher risk—colonoscopy yearly for extensive colitis with moderate-to-severe endoscopic or histological inflammation, CRC in a first-degree relative <50 years, PSC diagnosis, stricture or dysplasia in past 5 years in a patient who declines colectomy. 

In the latest “Endoscopic tissue sampling—Part 2: Lower gastrointestinal tract. European Society of Gastrointestinal Endoscopy (ESGE) Guideline” [57], optimal surveillance is achieved in case of endoscopic remission. This is crucial because distinguishing between dysplasia and inflammation based on endoscopic appearance and even mucosal biopsies becomes challenging in the case of active colitis. In high-risk patients, the combination of chromoendoscopy with targeted biopsies can be supplemented by non-targeted biopsies taken every 10 cm along the colon in all four quadrants [57]. In cases where there is the absence of endoscopic activity, advanced imaging technologies may prove beneficial in determining regions for targeted biopsies. This approach aims to evaluate histologic remission, especially when therapeutic implications are at stake [57]. If dysplasia (low- or high-grade) is detected without a corresponding endoscopically visible lesion, it is imperative to promptly conduct a repeat chromoendoscopy. This should be carried out by an experienced endoscopist to ascertain the presence of a well-circumscribed lesion and to evaluate the possibility of synchronous visible lesions [57]. Upon confirmation of low-grade dysplasia in absence of an associated endoscopically visible lesion, a follow-up step involves undergoing repeat chromoendoscopic colonoscopy with additional random biopsies within three months [57]. Finally, dysplasia confirmation should be sought from an independent pathologist specialized in gastrointestinal diseases [57].

### 4.2. Methods of Colonoscopy Surveillance

#### 4.2.1. Dye Chromoendoscopy

According to ESGE, utilizing pancolonic dye chromoendoscopy (DCE) or virtual chromoendoscopy (VCE) with targeted biopsies for any visible lesions during surveillance endoscopy in IBD patients is recommended [55]. Extensive evidence from clinical trials and real-life studies supports the superiority of chromoendoscopy over white-light endoscopy for dysplasia detection, regardless of operator skills or the disposal of high-resolution scopes [58,59,60,61,62]. According to the “Surveillance for Colorectal Endoscopic Neoplasia Detection and Management in Inflammatory Bowel Disease Patients” (SCENIC) consensus, HD-WLE is deemed superior to standard definition WLE, and DCE is recommended over WLE in the surveillance of IBD [17]. Additionally, in cases where DCE cannot be achieved, such as due to endoscopic activity or suboptimal bowel preparation, random quadrant biopsies every 10 cm are suggested [17]. New technological advancements, including optical and digital enhancement tools, have significantly enhanced the quality and precision for recognizing vascular and mucosal patterns. Thus, the aim of novel endoscopic imaging is to depict histological changes in suspected neoplastic lesions, inflammation, or healing, presenting as potential alternative to DCE in surveillance programs. The regular application of 0.1% methylene blue or 0.1–0.5% indigo carmine pancolonic chromoendoscopy along with targeted biopsies for surveillance in long-standing IBD is recommended from ESGE and SCENIC consensus [17,57]. It has been documented that DCE improves dysplasia detection reducing the need for biopsies. A randomized controlled trial demonstrated a 3.2-fold improvement in the comprehensive detection of dysplastic lesions in patients with UC when compared to WLE random biopsy sampling [63]. Further, Picco et al. [64] prospectively revealed that DCE with indigo carmine employed in the surveillance of long-standing UC outperforms white-light endoscopy (WLE) in terms of detecting dysplasia (21.3% vs. 9.3%; *p* < 0.05). Recently, a trial randomizing IBD patients to either HD-DCE with indigo carmine or HD-WLE demonstrated a significantly higher frequency of detecting dysplastic lesions in the HD-DCE group (17% vs. 7%; *p* < 0.05) [65].

#### 4.2.2. Virtual Chromoendoscopy

VCE is a proper substitute for evaluation of a superficial vascular and mucosal pattern; its efficacy has already been widely demonstrated in diagnostic endoscopy. By digitally post-processing endoscopic imaging, VCE amplifies the tissue surface detailing and aspires to depict histology with heightened accuracy. Existing technologies in the market comprise (1) Narrow Band Imaging (NBI) (Olympus, Tokyo, Japan): utilizes wavelengths absorbed by hemoglobin to maximize contrast; (2) iSCAN (Pentax, Tokyo, Japan): virtually enhances blue color to relatively dark areas for improved visualization; (3) Blue Laser Image (BLI) (Fujifilm, Tokyo, Japan): capable of evaluating both microvessels and the mucosal surface; (4) Linked Color Imaging (LCI) (Fujifilm, Tokyo, Japan): magnifies the color range between red and white, boosting subtle mucosal changes in case of inflammation and cancer. So far in IBD surveillance there is still inadequate data to suggest it as a favored method [7]. In a multicenter randomized controlled trial comparing DCE against NBI, similar results were shown in neoplasia and dysplasia detection [66]. A non-inferiority trial conducted by Iacucci and colleagues, comparing HD-WLE with HD-iSCAN and HD-DCE, revealed a difference not statistically significant in the neoplastic lesion detection rate [67]. Furthermore, according to the findings of the VIRTUOSO trial, iSCAN compared to HD-WLE showed no significant difference for detection of neoplasia in 188 IBD patients. The dysplasia detection rate was 24.2% for HD-WLE and 14.9% for VCE (*p* = 0.14) [68].

### 4.3. Endoscopic Classification of Visible Lesions

Following the SCENIC consensus, the designation “dysplasia-associated lesion or mass” (DALM) was discarded. The fundamental recommendation from guidelines and consensus for endoscopists is to distinguish dysplasia as invisible or visible. Paris classification categorized colonic lesions into polypoid and non-polypoid types. The polypoid lesions were further classified as either pedunculated or sessile. Among the non-polypoid lesions, subcategories include superficial elevated, flat, and depressed types. The “modified” Paris classification was introduced in IBD surveillance to define the morphology of the lesion and characterize the borders and the presence of an eventual ulceration [7]. In addition to gross morphology of colonic visible lesion (described according to Paris classification), endoscopy evaluates the surface pit pattern of the lesion, a characterization that can be defined using Kudo’s pit pattern [69]. Neoplastic pit patterns, from IIIs to Vi, were found to be a predictor of dysplasia even in IBD [62]. Incorporating magnification during colonoscopy could significantly improve the delineation of the pit pattern and enhance correlation with histology (both dysplasia and intramucosal carcinoma) [70]. Magnification facilitates a clearer distinction of lesion margins from the surrounding mucosa and enhances the differentiation of submucosal invasion in subtle lesions [71]. The latter introduced endoscopic classification for IBD lesions is the Frankfurt Advanced Chromoendoscopic IBD LEsions (FACILE) classification [72]. This fully validated classification departs from Kudo’s pit pattern and incorporated 4 key features to predict neoplastic lesions: (1) the morphology (nonpolypoid or polypoid); (2) the mucosal surface (roundish, villous, irregular/nonstructural); (3) vessels (nonvisible, regular, irregular/nonstructural); (4) any sign of inflammation. Due to this classification, trainees demonstrated a significant improvement in their capability to describe lesions, achieving a sensitivity and accuracy of 80% and 77%, respectively, after a specific training program (*p* < 0.001) [72].

## 5. Advanced Endoscopic Resection in Inflammatory Bowel Disease

The key to effectively manage CAN in IBD patients is to first identify them and subsequently guarantee a complete endoscopic resection (ER) in order to avoid surgery and opt for continuing surveillance. Both American and European guidelines recommend that only dysplasia that is visible can be endoscopically removed, although there is lack of strictly defined criteria for determining eligibility for ER of CAN [17,19]. The expertise of the specific endoscopist has a pivotal role in assessing the feasibility of ER and the probability of successful outcomes, including en bloc resection and R0 resection. Consequently, it is highly advisable to consider referring any patient with CAN to proficient IBD and therapeutic advanced endoscopy centers, led by operators skilled in ER. 

**Figure 2:**
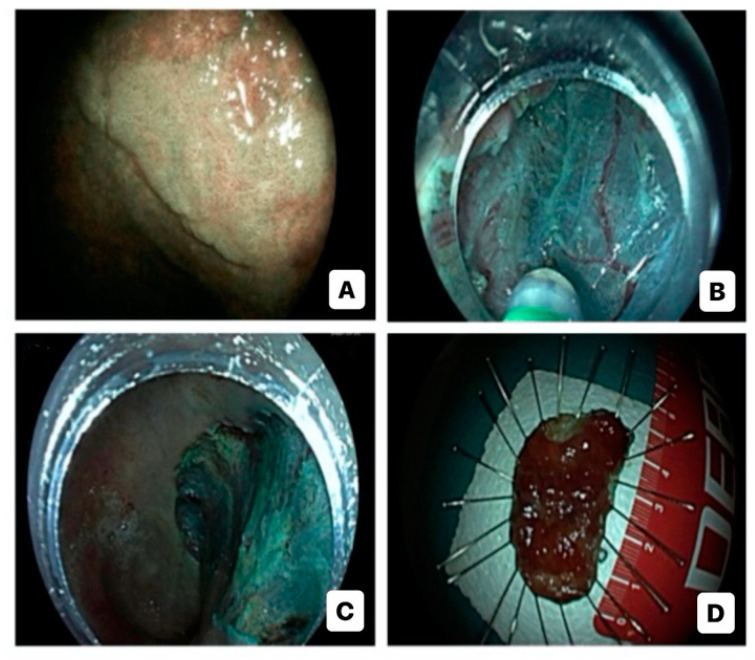
Endoscopic submucosal dissection for high-risk colitis-associated colorectal neoplasia: virtual chromoendoscopic evaluation (**A**); dissection (**B**); post-dissection inspection (**C**); specimen (**D**).

Advanced endoscopic resection techniques for CAN include endoscopic mucosal resection (EMR), endoscopic submucosal dissection (ESD), and hybrid ESD-EMR (h-ESD) approach (Figure 2). Support for their use comes from three meta-analyses which underline that advanced ER is effective and safe in the management of large dysplastic lesions in IBD, although a close endoscopic monitoring due to the risk of local recurrence (2–5 cases per 1000 person-years of follow-up) and metachronous dysplasia is needed [73,74,75]. Nevertheless, ER of CAN, especially with lesions >20 mm, can be quite difficult, primarily due to persistent activity, scarring, and fibrosis, caused by the recurrence of inflammation–healing cycles [20]. Numerous studies have shown that performing EMR and ESD in the context of CAN is both safe and feasible [76,77,78,79,80,81,82,83,84,85,86,87,88,89,90]. These studies, although relatively small and retrospective, collectively involved more than 500 patients for a total of almost 600 lesions. The majority of these patients were diagnosed with UC. Out of these studies, ten exclusively focused on ESD procedures, while five others provided information on both ESD and EMR procedures or hESD. 

In a recent 3-round modified international Delphi consensus, the term CAN was suggested for “all neoplastic lesions detected in a section of previously or presently inflamed colon” [91]. Neoplastic lesions found in regions of the colon that have not been previously affected by inflammation were classified as sporadic and were not associated with colitis [92]. According to this consensus, nonpolypoid lesions and large (>20 mm) nonpedunculated polyps should be classified as high-risk CAN (HR-CAN) [91]. Indeed, nonpolypoid lesions were considered as an independent risk factor for advanced neoplasia in IBD, while large nonpedunculated polyps were considered to have a heightened risk of progressing into submucosal invasive cancer [93,94,95]. An HR-CAN was considered endoscopically resectable if (a) there were distinct margins; (b) it could (preferably) be removed en bloc with clear margins; (c) there was no confirmation of synchronous invisible dysplasia; (d) there was no evidence of moderate-severe inflammation of mucosa surrounding the area with HR-CAN; (e) deep submucosal invasion signs were absent [91]. HR-CAN demands a comprehensive evaluation that adheres to a standardized protocol, including (1) size, delineation, and location; (2) gross morphology; (3) pit and vascular pattern at chromoendoscopy; (4) endoscopic activity in the affected segment, harboring the dysplastic lesion [91].

For HR-CAN, the preferred approach is to remove it en bloc, as this reduces the risk of recurrence and optimizes histopathological evaluation [91]. Performing ESD in IBD patients can be more challenging compared to non-IBD settings due to several factors related to the nature of IBD and its impact on the gastrointestinal tract. In IBD, chronic inflammation in the gastrointestinal tract leads to changes in the mucosal and submucosal layers with a resulting fibrosis. The repeated inflammation and the consequent fibrosis reduce the submucosal space, decreasing the possibility of accessing to the third space. As a result, visualization of the cleavage planes is compromised, and the risk of perforation may increase. Further, inflammatory changes can lead to a difficult demarcation of the target lesion and surrounding tissue. Therefore, the use of traction might be beneficial for the creation of triangulation and improvement in the feasibility of the technique in the IBD setting. 

A recent meta-analysis found that ESD or h-ESD achieved pooled en bloc and R0 resection rates of 86% and 70%, respectively, in the case of nonpolypoid lesions [96]. The pooled recurrence rate was 8% [96]. Notably, ESD had significantly (*p* < 0.001) higher en bloc resection rates (93%) compared to h-ESD (65%) [96]. Similarly, ESD had higher pooled R0 resection rates (75%) compared to the hybrid technique (60%), although the difference did not reach statistical significance (*p* = 0.454) [96]. Data on the outcomes of piecemeal resection are varied. While piecemeal EMR has demonstrated outstanding early and long-term results for sporadic adenomas larger than 20 mm [97], piecemeal resections of sporadic nonpolypoid lesions have been associated with a pooled recurrence rate of 20%, compared to 3% of en bloc resections [98]. 

In larger polyps (greater than 20 mm), the recurrence rate can even exceed 30% [99]. However, recent advances in the use of snare-tip soft coagulation on the resected lesion margin have significantly reduced the risk of recurrence after a piecemeal EMR. It is essential to highlight that the applicability of these findings to the setting of CAN is unclear, as there is a lack of studies in this specific population. 

In a recent retrospective, multicenter, eastern cohort study involving UC patients who experienced ER for CAN, 142 lesions were managed with EMR, and 96 lesions were treated using ESD [100]. En bloc resection and AEs were 92.8% and 2.5%, respectively, in 238 patients, demonstrating promising outcomes of ER for neoplasia in patients with UC [100]. Follow-up was conducted for 146 lesions, revealing a local recurrence rate of 2.7% [100]. Furthermore, metachronous neoplasia rate following ER was 6.1% [100]. 

Kaltenbach et al. [101] retrospectively included 326 IBD patients who underwent surveillance endoscopy. The rate of nonpolypoid colorectal lesions was 7.7% (63 lesions) with a mean size of 17.8 ± 8.9 mm, ranging from 10 to 45 mm [101]. Pathological findings revealed 3 cases of high-grade dysplasia, 27 cases of low-grade dysplasia, 14 cases of sessile serrated lesions, 6 hyperplastic lesions, and 13 inflammatory lesions [101]. The findings support ER (including EMR, ESD, or standard technique) and surveillance colonoscopy as a safe and effective management for IBD patients with nonpolypoid colorectal dysplasia [101]. Endoscopic resection of nonpolypoid colorectal lesions was found to be feasible (with a success rate of 96.8%) and associated with a low incidence of AEs (1.5%) [101]. Moreover, in this study, the authors provide valuable long-term outcome data, demonstrating a low rate of recurrence (6.3%; 95% CI, 1.8–15.5) [101]. 

Recent findings from a real-world multicenter Italian study, evaluating safety and effectiveness of ESD for HR-CAN in long-standing IBD patients, included 90 lesions [102]. ESD and hESD were performed in 82% and 18% of cases; en bloc resection and R0 resection were completed in 97% and 86% of cases [102]. Regarding adverse events (AEs), 29% of cases experienced them [102]. Further, after 2 years, three local recurrence and three metachronous lesions occurred [102]. Furthermore, similar data were shown in a retrospective multicenter study including all consecutive ESD in IBD patients with visible dysplasia (88 lesions) in French centers [103]. En bloc resection, R0 resection and curative resection were achieved in 80 (91%), 72 (82%) and 70 (80%) lesions, respectively [103]. Surgery was required in 1.2% of cases for complication, in 3.6% of cases for technical failure and in 7.3% of cases for bad prognostic histological features [103]. Local recurrence rate was lower in the high-volume centers compared with low-volume centers (0% vs. 9.3%, *p* = 0.118) and higher in CD patients than in UC patients (15.8% vs. 1.6%, *p* = 0.038) [103]. 

A summary of the main studies described in the literature is specified in Table 1.

However, to date, there have been no studies conducted to determine the most effective follow-up strategy following the endoscopic removal of CAN. Guidelines endorsed by ASGE and ESGE suggest that patients with IBD should undergo endoscopic surveillance between 3 and 6 months after a complete ER. If no recurrence is detected during surveillance, it is recommended to schedule a follow-up colonoscopy after one year. In cases where the resection was performed piecemeal or when positive lateral margins were found without the need for surgery, a colonoscopy with biopsy sampling is recommended at 3 months. 

For multifocal dysplastic lesions, surgical resection is recommended. Patients presenting with high-grade dysplasia (HGD) in the context of non-demarcated (or invisible) lesions may face an elevated risk of concurrent CRC, ranging from 75% to 100% for singular or multifocal lesions, respectively [104]. Consequently, in cases where lesions are deemed invisible, incompletely resected, or not amenable to ER, surgery should be strongly considered [104]. The standard procedure for cases involving HGD or CRC is total proctocolectomy with ileal pouch-anal anastomosis [105]. In cases of patients with significant comorbidities, endoscopically unresectable unifocal neoplasia without other high-risk histological factors, and colonic CD without rectal involvement, a subtotal or partial colectomy might be considered as an optional treatment [105]. 

## 6. Conclusions

IBD patients face a twofold higher risk of developing CRC in comparison to the general population. IBD-CRC follows a distinct genetic and molecular pathogenesis compared to sCRC and can be considered a complication caused by the recurrence of chronic intestinal inflammation. The decreased incidence of IBD-CRC can be attributed to improved therapeutic management of inflammation, advancements in endoscopy techniques, and early detection of precancerous lesions through surveillance programs. The use of advanced imaging technologies has rendered previously undetectable dysplasia visible in most cases, enabling a more precise and detailed examination of the mucosa. Furthermore, new tools have facilitated the ER of visible lesions, allowing patients a possibility to avoid colectomy. In this context, ESD, when performed in tertiary endoscopy centers, was a feasible, safe, and effective strategy to manage CAN in patients with IBD, with low rate of local recurrence and metachronous lesions considering short-term follow-up. However, further prospective studies including long-term follow-up are still required to highlight the impact of ESD for IBD patients’ dysplasia-free survival.

## Figures and Tables

**Table 1:** Main outcomes of endoscopic submucosal dissection for colitis-associated colorectal neoplasia in IBD patients.

First Author, Year	Lesions, n, Study Design	En bloc	R0	AEs	Local Recurrence	Methacronous Lesions	Post-Dissection Surgery	Follow-Up, Months
Manta 2021 [90]	53, Case Series	53/53 (100%)	51/53 (96.2%)	10/53 (18.9%)	0/53 (0%)	2/53 (3.8%)	2/53 (3.8%)	37 (6–60)
Yang 2019 [85]	15, Retrospective Study	14/15 (93.3%)	12/15 (80%)	0/15 (0%)	2/15 (13.3%)	2/15 (13.3%)	0/15 (0%)	24 (5–64)
Kochhar 2018 [84]	7, Case Series	6/7 (85.7%)	6/7 (85.7%)	0/7 (0%)	0/7 (0%)	0/7 (0%)	1/7 (14.3%)	6
Suzuki 2017 [82]	32, Retrospective Study	29/32 (91%)	23/32 (71.8%)	1/32 (3.1%)	1/32 (3.1%)	3/32 (9.4%)	4/32 (12.5%)	33 (6–76)
Iacopini 2015 [80]	10, Case Series	8/10 (80%)	8/10 (80%)	1/32 (3.1%)	2/10 (20%)	3/10 (30%)	1/10 (10%)	24 (6–72)
Ngamruengphong 2022 [79]	45, Retrospective Study	43/45 (95.6%)	34/45 (75.6%)	5/45 (11.1%)	1/45 (2.2%)	11/45 (24.4%)	3/45 (6.7%)	18 (13–37)
Lightner 2021 [78]	25, Retrospective Study	23/25 (92%)	22/25 (88%)	1/45 (2.2%)	0/25 (0%)	3/25 (12%)	10/25 (40%)	19 (7–53)
Kasuga 2021 [77]	11, Retrospective Study	10/11 (90.1%)	9/11 (82%)	3/11 (27%)	0/11 (0%)	2/11 (18.2%)	1/11 (9.1%)	25 (1–132)
Hirai 2023 [100]	96, Retrospective Study	94/96 (96.9%)	88/96 (91.7%)	6/96 (6.3%)	2/96 (2%)	6/96 (6.2%)	NA	35
Anneraud 2023 [103]	88, Retrospective Study	80/88 (91%)	72/88 (82%)	13/88 (14.8%)	4/88 (4.5%)	NA	10/88 (11.4%)	26 ± 25
Maselli 2023 [102]	90, Retrospective Study	87/90 (97%)	77/90 (86%)	26/90 (29%)	3/90 (3%)	3/90 (3%)	13/90 (14%)	24

## Data Availability

Not applicable.
